# Bronchial obstruction in osteogenesis imperfecta can be detected by forced oscillation technique

**DOI:** 10.3389/fmed.2023.1301873

**Published:** 2023-12-21

**Authors:** Silvia Storoni, Sara J. E. Verdonk, Dimitra Micha, Patrick M. C. Jak, Marianna Bugiani, Elisabeth M. W. Eekhoff, Joost G. van den Aardweg

**Affiliations:** ^1^Department of Internal Medicine Section Endocrinology and Metabolism, Amsterdam UMC Location Vrije Universiteit, Amsterdam, Netherlands; ^2^Amsterdam Rare Bone Disease Center, Amsterdam Movement Sciences, Amsterdam, Netherlands; ^3^Department of Human Genetics, Amsterdam UMC Location Vrije Universiteit, Amsterdam, Netherlands; ^4^Department of Respiratory Medicine, Amsterdam University Medical Center, Location AMC, Amsterdam, Netherlands; ^5^Department of Pathology, Amsterdam University Medical Centre, Location VUmc, Amsterdam, Netherlands

**Keywords:** osteogenesis imperfecta (OI), forced oscillation technique (FOT), bronchial obstruction, lung function, collagen type 1

## Abstract

**Introduction:**

Respiratory insufficiency is a leading cause of death in individuals with osteogenesis imperfecta (OI). However, evaluating pulmonary function in OI presents challenges. Commonly used pulmonary function tests such as spirometry and body plethysmography are sometimes difficult to perform for OI patients, and reference intervals are not always applicable. The forced oscillation technique (FOT) is a patient-friendly method for detecting respiratory abnormalities that requires no effort from the patient.

**Objective:**

This study investigates the feasibility of FOT in the evaluation of respiratory function in the clinical management of OI patients.

**Methods:**

Twelve OI patients, comprising eight with Sillence OI I, two with OI IV, and two with OI III, underwent spirometry, body plethysmography, and FOT, both pre-and post-administration of salbutamol.

**Results:**

FOT measurements exhibited consistent trends that aligned with spirometry and body plethysmography findings. The resistance at 8 Hz decreased after the administration of salbutamol, indicating that FOT is able to detect bronchial obstruction and its alleviation by medication (*p* < 0.05). The resonant frequency during expiration was higher than during inspiration in nearly all patients, suggesting obstructive disease. The technique gives insight into both inspiratory and expiratory impairment of pulmonary ventilation. The main FOT parameters showed a relatively high repeatability in duplicate measurements.

**Conclusion:**

Bronchial obstruction can be detected by FOT in patients with OI during quiet breathing, making it an easily executable alternative to other lung function measurements. The technique can detect the bronchodilator effect of sympathomimetic medication. It has the potential to provide information on expiratory flow limitation, pulmonary restriction, and reduced lung compliance.

## Introduction

Respiratory complications are a major cause of death in patients with osteogenesis imperfecta (OI), occurring three times more frequently than in the reference population (1, 2). OI is a genetic connective tissue disorder characterized by bone fragility and bone deformities caused by pathogenic variants in genes responsible for the proper formation of collagen type I (3–6). Patients’ phenotypes range from non-deforming OI to progressively deforming OI, in which patients are frequently wheel-chair bound, to perinatally lethal (7–9). In addition to skeletal abnormalities, OI patients may experience complications such as hearing loss, dentinogenesis imperfecta, cardiovascular problems, and respiratory complications (10–14). Despite the evident significance of respiratory complications in OI, there are currently no established guidelines for the management and monitoring of respiratory function in OI patients. This knowledge gap appears to be partially attributed to a limited understanding of the underlying pathology.

To date, the pulmonary function tests (PFTs) that are mostly used to evaluate the respiratory function in patients with OI are spirometry and body plethysmography (15). However, both tests pose limitations in the OI population (16). The measurement of forced expiratory lung volume in spirometry can be difficult to perform for OI patients as it requires forced breathing (17, 18). The body plethysmography necessitates the patient to sit in an airtight chamber. This may turn out to be challenging for the OI patients who are wheel chair-bound (as well as for patients with claustrophobia) (19, 20). Both tests reference intervals have been calculated using healthy individuals, and they are standardized based on height, arm span, and weight. Due to bone deformities and fractures, patients with OI typically have a lower-than-average height, causing a discrepancy between height and arm span that is not found in the reference population. Additionally due to bone deformities and fractures, OI patients may have chest deformities. This raises the question of what the results of these examinations can reliably reveal about respiratory function in patients with OI (21). Thus, even though spirometry and body plethysmography are the gold standards for evaluating respiratory function, they have limitations in OI.

Despite the fact that respiratory issues are one of the leading causes of death in patients with OI, pulmonary issues are not commonly diagnosed in this population. The unsuitability of commonly used PFTs for patients with OI may mean that respiratory issues often go undetected and are not diagnosed until the condition has become severe. This study aims at applying a pulmonary function test that may be more suitable to use for detecting respiratory abnormalities in patients with OI. Forced oscillation technique (FOT) is a non-invasive method for assessing lung function (22). The test consists of breathing through a mouthpiece while being exposed to oscillating airflow at different frequencies. The test takes less than 5 min and requires no effort from the patient, who has to breathe normally through the mouthpiece (23, 24). The FOT test provides a comprehensive assessment of both central and peripheral airways, making it a useful tool for detecting respiratory abnormalities in patients with various lung diseases (23, 25, 26). The mechanical impedance spectrum of the respiratory system has distinct, frequency-dependent properties that are relatively sensitive to the development of lung disease (27). The aim of this study was to assess the feasibility of FOT in evaluating respiratory function for the clinical management of patients with OI.

## Methods

### Study population

Twelve patients diagnosed with OI were included in this study. All participants provided informed consent for the utilization of their medical data. The study aimed at analyzing patients with OI regardless of preexisting pulmonary conditions. The patients’ phenotype was determined using the Sillence classification; non deforming OI (OI type I), moderate OI (OI IV) and progressively deforming OI (OI type III). No exclusion criteria were applied.

### Study procedures

Patients underwent spirometry with diffusion testing (Masterscreen PFT, Vyaire, Germany), body plethysmography (Masterscreen Body, Vyaire, Germany), and FOT. Patients performed these measurements both before and after the administration of 400 μg of salbutamol. FOT measurements were performed twice before and twice after salbutamol administration. This approach aimed to test the repeatability of the collected data. During the examination, patients were instructed to maintain an upright sitting position with their backs straight. They were asked to place their hands on their cheeks and chin to minimize the dissipation of the imposed oscillations. Patients were instructed to breathe normally throughout the 90 seconds (90 s) test duration.

### Forced oscillation technique

**Figure 1:**
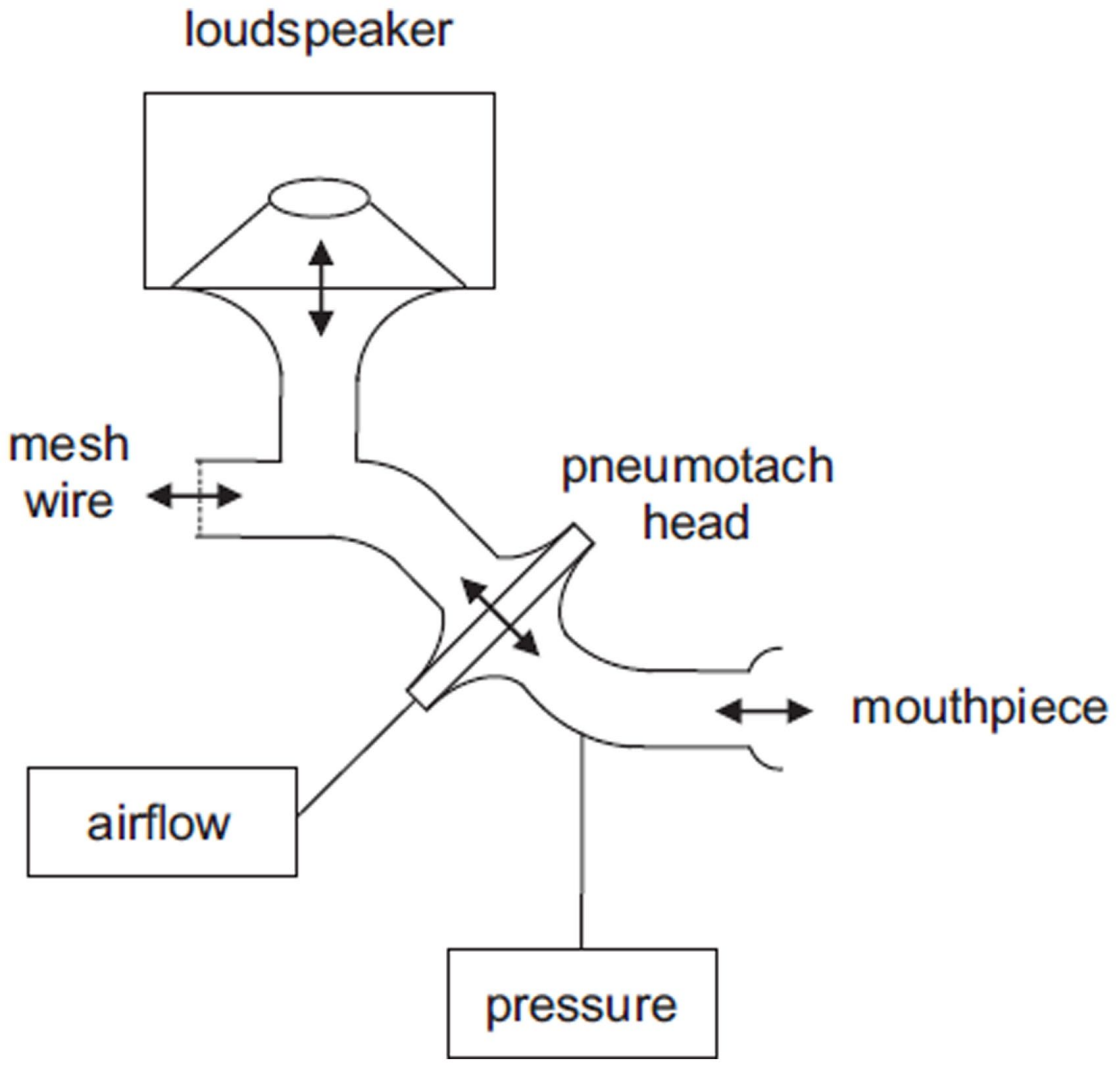
Schematic drawing of the FOT device. The patient breathes through the mouthpiece and the mesh wire. The loudspeaker generates oscillations that are transferred towards the mouthpiece. Pressure is measured in the mouthpiece and airflow is derived from the pressure difference across the pneumotach head.

Respiratory system impedance was continuously measured using a modified forced oscillation device. A schematic drawing of the FOT device is shown in Figure 1. Oscillations were applied with a loudspeaker connected to a mouthpiece. The respiratory impedance was derived from the relation between induced oscillations in airflow and pressure, measured in the mouthpiece. The device consisted of a modified Masterscreen IOS system (Vyaire Medical, Germany), where the loudspeaker was controlled by a computer through a digital-to-analog and analog-to-digital converter (USB-6212, NI, Texas). The airflow was measured with the Masterscreen IOS system. The pressure was measured with a Hans Rudolph Pneumotach Amplifier I (Hans Rudolph, Kansas). The loudspeaker generated simultaneous oscillations of 8, 12, 16, 20, and 24 Hz with an amplitude of approximately 1.0 cmH_2_O. Calibration of the device was conducted using a calibration device with a standard resistance (Vyaire Medical, Germany). During the FOT, patients breathed through an antibacterial filter with an approximate resistance of ~0.3 cmH_2_O s/L at the prevailing airflows (Microgard II, Vyaire, Germany). Analog pressure and flow signals were digitized at 800 Hz. The estimation of time-and frequency-dependent respiratory impedance (Zrs) was based on the assumption that random errors occurred in both pressure and flow. This resulted in a bivariate least squares estimate of respiratory impedance as a function of time and frequency. Confidence intervals were also calculated as a function of time and frequency. Further details regarding this modeling approach can be found in a patent application form (US 2012/0289852 A1).

### FOT parameters

The FOT was employed to examine the following parameters: resonant frequency and mean resistance and reactance at 8, 12, 16, 20, and 24 Hz. These were determined separately during inspiration and expiration. The absence of established reference intervals for FOT hinders the establishment of a definitive normal range due to device-based data variations. However, analyzing disparities in values between inspiration and expiration, as well as pre-and post-therapy administration, provides crucial insights. The impedance of the respiratory system (Zrs) describes the linear relationship between pressure and flow variations during oscillatory airflow in and out of the lungs. Resistance (Rrs) is the in-phase component of Zrs and is related to frictional resistance. Rrs reflects the resistance to changes in airflow and is a measure of the degree of lung obstruction. Reactance (Xrs) is the 90° out-of-phase component of Zrs and comprises both inertance and elastance. Inertance is mainly related to the mass of accelerating air in the airways. Elastance quantifies the stiffness of the entire respiratory system, including the chest wall, lungs, and airway walls, depending on the mechanical properties of these structures. The resonant frequency (ƒres) is measured in Hz and represents the frequency at which pressure and flow are perfectly in phase. In healthy individuals, the resonant frequency is typically around 8 Hz.

### Statistical analysis

The data presented in this article are primarily descriptive in nature, aiming at exploring the potential utility of this method for lung function detection in OI patients. The Wilcoxon test was utilized to determine the significance of differences observed before and after salbutamol administration. Pearson correlation coefficients were calculated to investigate potential correlations between parameters of the FOT and results of the other pulmonary function tests. The repeatability for several FOT parameters (the mean overall impedance at 8 Hz during inspiration and expiration, and the mean resonant frequency during inspiration and expiration) was assessed using the Bland–Altman analysis (28), on the basis of repeated measurements. Between the two measurements before or after salbutamol administration, the more consistent FOT measurement was selected for analysis.

## Results

Twelve patients clinically and genetically diagnosed with OI were included in the study. Based on the Sillence classification, eight patients had OI type I, two had OI type IV, and two had OI type III. The study population consisted of seven females and five males. The median age of the patients was 49 years (standard deviation 14). All patients underwent spirometry and FOT measurements. Two patients refused to undergo the body box plethysmography test due to fear or anxiety associated with the procedure. None of the patients had a history of chronic obstructive pulmonary disease, and only one patient had a diagnosis of asthma. Only one patient reported a history of smoking. Among the patients, six had scoliosis of varying degrees, of whom two were wheelchair-bound due to the severity of their condition. The Tiffeneau index and forced vital capacity (FVC), obtained from spirometry before and after the administration of salbutamol, are presented in Table 1. Mild obstruction was found using spirometry or plethysmography in patients 01, 02, 07 and 08 (Table 1).

**Table 1:** Results of the FOT prior to and after administration of salbutamol.

		FOT												Spirometry		
		PRE		POST		PRE		POST		PRE		POST		PRE-POST	PRE-POST	PRE-POST
Pt (n)	Sex	ƒresIn	ƒresEx	ƒresIn	ƒresEx	RrsIn08	RrsEx08	RrsIn08	RrsEx08	XrsIn08	XrsEx08	XrsIn08	XrsEx08	Tiffeneau-index (%)	FVC (L)	FEV1 (L)
01	F	6.55	8.22	5.40	5.94	3.33	4.80	3.25	4.64	0.16	−0.06	0.37	0.46	67–80	2.5–2.6	1.80–2.11
02	M	5.35	9.67	4.85	8.43	2.83	6.49	2.76	6.95	0.34	−0.11	0.41	0.27	78–81	4.0–4.1	3.15–3.28
03	M	8.59	8.59	5.46	4.95	3.27	3.79	2.51	3.06	−0.05	0.11	0.45	0.66	84–87	4.4–4.5	3.65–3.86
04	M	7.15	5.49	3.15	1.51	3.59	3.29	2.57	2.47	0.15	0.45	0.65	0.78	78–79	4.2–4.6	3.44–3.67
05	F	8.05	7.32	7.94	5.76	3.45	4.09	3.02	3.27	−0.16	0.11	−0.13	0.31	87–85	3.2–3.2	2.75–2.71
06	F	5.32	7.48	4.90	2.92	2.55	4.22	2.14	2.55	0.34	0.21	0.28	0.58	79–80	4.3–4.3	4.43–3.56
07	F	8.08	23.97	8.55	15.53	4.74	9.90	4.92	7.29	−0.24	−12.31	−0.23	−3.18	63–87	1.5–1.7	1.21–1.53
08	M	10.51	16.14	8.44	10.29	2.90	5.36	2.99	5.26	−0.57	−1.91	−0.31	−0.51	83–85	2.1–2.1	1.77–1.80
09	F	5.49	4.17	4.21	4.02	2.76	2.88	2.62	3.42	0.26	0.46	0.39	0.62	84–87	4.1–40	3.46–3.49
10	F	5.45	4.10	4.99	3.36	2.15	2.44	2.04	2.40	0.27	0.51	0.28	0.55	71–70	3.4–3.5	2.36–2.48
11	F	9.29	9.66	5.91	5.98	4.41	4.96	3.35	3.70	−0.27	−0.23	0.37	0.51	69–75	3.5–3.6	2.42–2.70
12	M	9.52	8.83	3.86	2.40	3.39	3.26	2.17	2.35	−0.21	−0.03	0.55	0.77	74–78	4.4–4.4	3.27–3.42

Pt, patient; F, female; M, male; ƒresIn, resonant frequency during inspiration; ƒresEx, resonant frequency during expiration; RrsIn08, resistance during inspiration at 8 Hz; RrsEx08, resistance during expiration at 8 Hz; XrsIn08, reactance during inspiration at 8 Hz; XrsEx08, reactance during expiration at 8 Hz; PRE and POST salbutamol administration; FVC, forced vital capacity; FEV1, forced expiration volume in 1 s.

**Figure 2:**
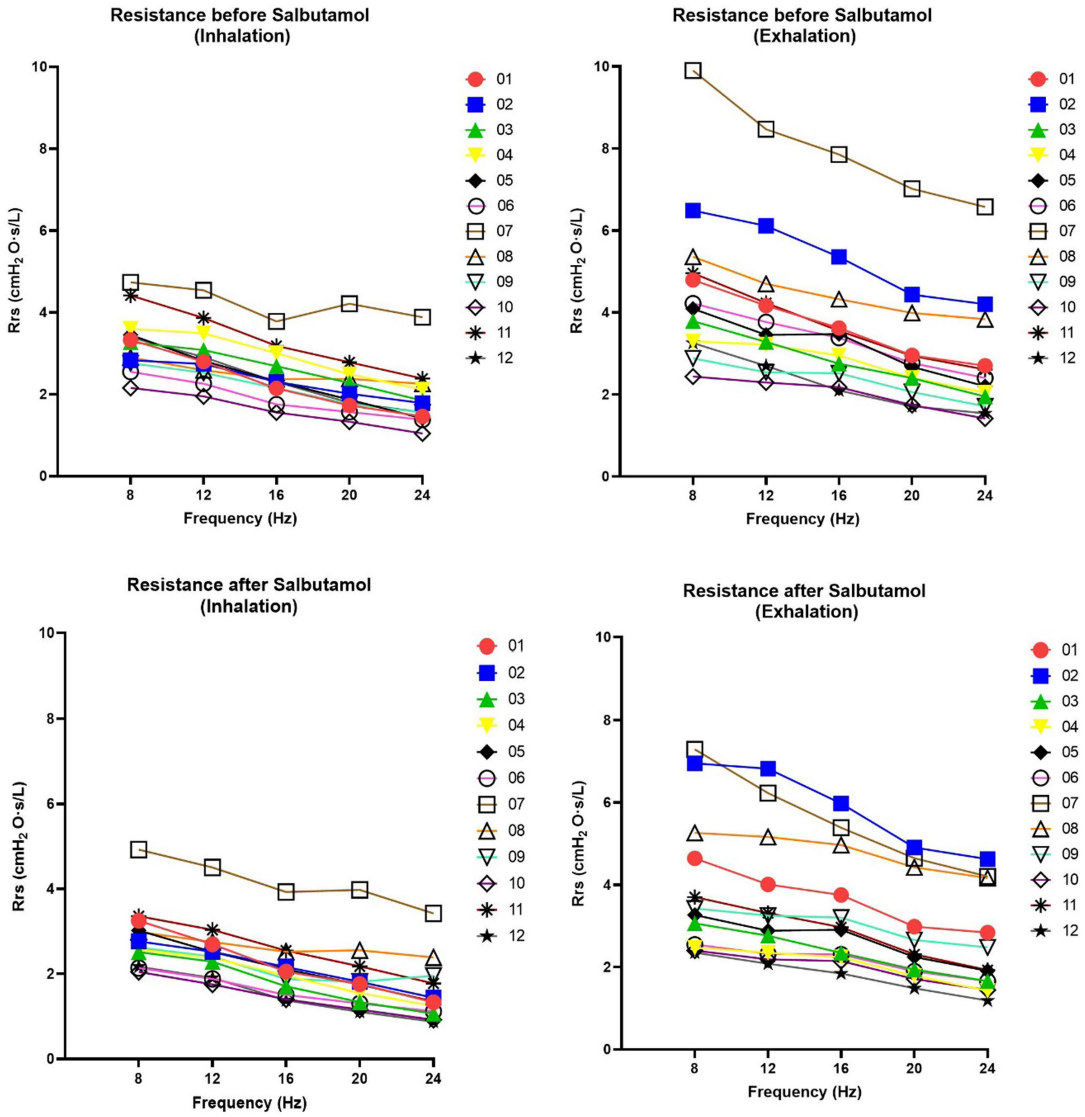
Changes in resistance of respiratory system (Rrs) at 08, 12, 16, 20 and 24 Hz. Rrs was assessed before and after salbutamol administration. Graphs on the left show Inspiration phases whereas graphs in the right show expiration phases. A decrease after salbutamol administration (in particularly noted during the exhalation) is indicative for a bronchial obstructive pattern. Rrs demonstrates a linear relationship with frequency across all patients and under all conditions (*r*^2^ > 0.95).

**Figure 3:**
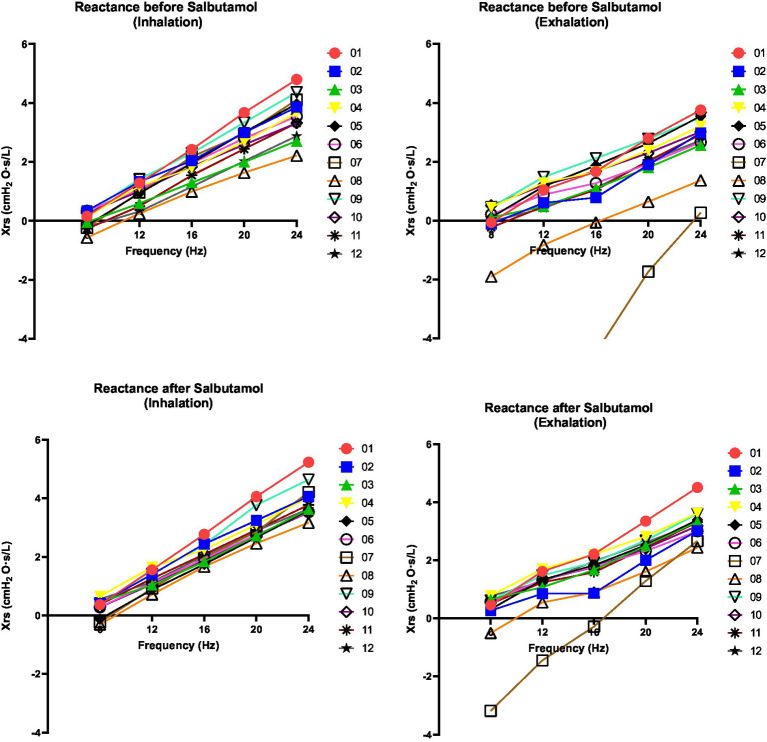
Changes in reactance of respiratory system (Xrs) at 08, 12, 16, 20 and 24 Hz. Xrs was assessed before and after salbutamol administration. Left graphs indicate inspiration phases whereas right graphs indicate expiration phases. A decrease after salbutamol administration (in particularly noted during the exhalation) is indicative for a bronchial obstructive pattern. Xrs demonstrates a linear relationship with frequency across all patients and under all conditions (*r*^2^ > 0.95).

The results of the FOT parameters before and after the administration of 400 μg of salbutamol are presented in Table 1. Among the 12 patients, eight exhibited higher resonant frequency values during expiration compared to inspiration. Following salbutamol administration, the resonant frequency during inspiration decreased in 11 patients (*p* = 0.006). In all patients, the resonant frequency during expiration decreased after salbutamol administration (*p* = 0.002). Regarding resistance, after the administration of salbutamol, 10 patients experienced a reduction in resistance during inspiration, while 11 patients showed a decrease in resistance during expiration (*p* = 0.019 for both comparisons). After salbutamol administration, the reactance increased in all patients during both inspiration and expiration (*p* = 0.006 and 0.019, respectively). Resistance decreased with higher frequencies, while reactance increased (Figures 2 and 3).

The difference in forced expiratory volume in 1 sec (FEV1), measured using spirometry, exhibited a slight correlation with the variance in resistance measured through FOT before and after salbutamol administration (Pearson Correlation 0.39; *p* = 0.021). The association between the relative ΔFEV1 and the relative Δ resonant frequency was found to be weak (Pearson Correlation 0.24; *p* = 0.46).

Table 2 shows the repeatability parameters for the overall impedance of the respiratory system (Zrs) at 8 Hz, as well as the resonant frequency, during both inspiration and expiration. These values were derived for duplicate 90 s measurements. The FOT parameters were computed as the mean of breath-to-breath values from each 90 s measurement. As indicated by the table, the standard deviations were relatively small and none of the parameters showed significant bias (the mean differences were not significantly different from zero at the 5%-significance level).

**Table 2:** Repeatability of FOT parameters, 90 s, 90 seconds test duration.

Parameter	Phase of the respiratory cycle	Duplicate 90 s measurements	
		Mean difference	Standard deviation
Mean Zrs at 8 Hz (cmH_2_O s/L)	Inspiration	0.14	0.57
	Expiration	0.11	0.56
Mean resonant frequency (Hz)	Inspiration	0.01	0.81
	Expiration	−0.16	1.91

## Discussion

Respiratory complications are a major cause of death in patients with OI. McAllion et al. (2) first reported this in 1996, but the topic received limited attention. Only recently, Folkestad et al. (29) investigated OI patient mortality in a large Danish cohort, finding a threefold higher risk of respiratory-related death than in the general population. The cause of OI pulmonary complications is debated, with some attributing it to restrictive lung function from skeletal abnormalities like scoliosis and rib fractures (30, 31). However, the Danish study linked respiratory-related deaths to chronic obstructive pulmonary disease (29). Our recent study on OI type II fetuses revealed intrinsic lung alterations, suggesting similar changes in other OI types (32). Our understanding of the mechanisms behind respiratory complications in OI remains limited. Evaluating pulmonary function in OI patients requires considering the limitations of current gold standard tests like spirometry and body box plethysmography. These tests require cooperation of the patient and are preferably performed in the upright position, which may not always be feasible for wheelchair-bound individuals or patients with skeletal deformities. Additionally, reference values may not be applicable to OI patients due to discrepancies between height and arm span. Could these factors contribute to underdiagnosis? The answer remains elusive. We propose FOT as a patient-friendly alternative for accurately measuring respiratory obstruction and restriction. FOT already proved to be a simple, sensitive, specific, and noninvasive technique (33).

**Figure 4:**
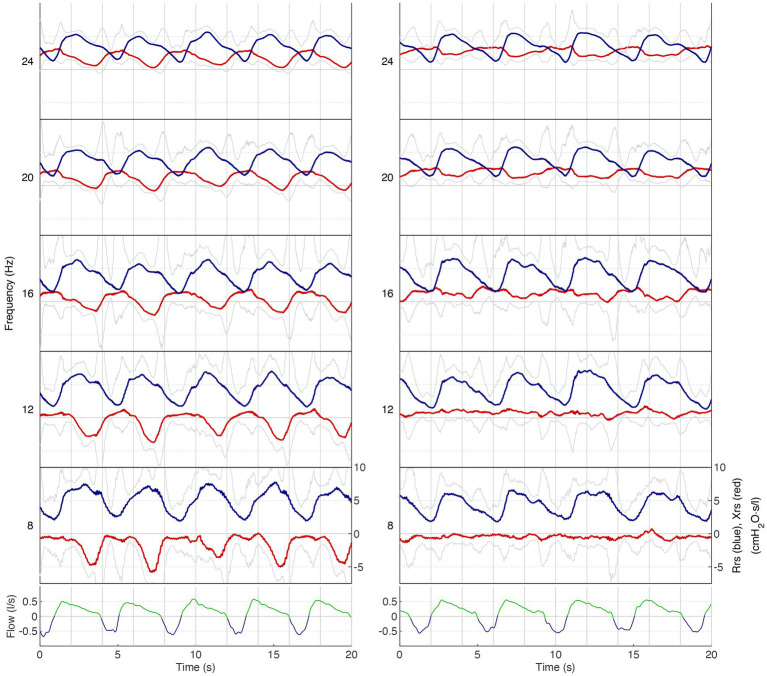
Respiratory impedance as a function of time for different frequencies in patient 8. Lower panel: airflow (low-pass filtered): blue, inspiration; green, expiration. Upper panel: respiratory impedance for five frequency bands (8–24 Hz): blue, Rrs; red: Xrs. Grey lines indicate the upper and lower limits of the 90%-confidence intervals of Rrs and Xrs, respectively. On the left is the measurement before salbutamol administration, and on the right is the measurement after salbutamol administration. Note that Rrs significantly increases during expiration while Xrs decreases. Especially the drop in Xrs during expiration indicates expiratory flow limitation (left panel), which is largely abolished after salbutamol (right panel).

Although we cannot draw definitive conclusions, our FOT measurements exhibited consistent trends that align with the findings of spirometry and body box plethysmography (Table 1). Notably, we observed a decrease in almost all resistance values following salbutamol administration (Table 1 and Figure 2). While the physiological ranges of these measurements remain unknown, it is interesting to note that this pattern was particularly evident in patients exhibiting an obstructive pattern on spirometry (Table 1). We observed that the resonant frequency during expiration was higher than during inspiration in nearly all patients (Table 1). This agrees with intrathoracic airway obstruction which increases during expiration (34, 35). The marked negative swings in Xrs during expiration that occurred in several patients indicates expiratory flow limitation (33). See, for instance, Figure 4, where these negative expiratory swings in Xrs were abolished by the administration of salbutamol. There are indications that the reactance at low frequencies becomes more negative in restrictive lung diseases during inspiration, when the lung is stretched to a greater extent (34). However, in obstructive diseases, the reactance tends to become more negative during expiration, when intrathoracic airway obstruction becomes more severe (33). Unlike previous studies where it was not possible to differentiate between a restrictive and an obstructive problem using FOT (36), our technique was able to assess the resistance, reactance, and the resonant frequency during both inspiration and expiration, giving insights in the respiratory function. Our findings provide support for the presence of obstructive lung disease in OI patients, as observed in four patients through standard PFTs and nearly all patients using FOT (16, 32). These results are consistent with the recent Danish study that emphasized obstructive disease as a significant contributor to hospitalization, medical utilization, and mortality in patients with OI (29). Taken together with our finding, this suggests a potential concomitant obstructive pulmonary disease alongside the well-known restrictive pulmonary disease in patients with OI (30).

This study presents a novel approach to the early identification of lung complications in OI, but it is not without limitations. As a feasibility study, the main limitation is the small sample size, which limits the ability to draw definitive conclusions. A larger cohort would be necessary to establish more robust findings. Additionally, the lack of standardized devices used in previous FOT studies poses challenges in directly comparing the results with existing literature. Further studies are needed to validate the findings and establish the diagnostic utility of FOT in identifying lung complications in OI. However, despite these limitations, this study represents the first attempt to apply a novel method for early identification of lung complications in OI patients, providing valuable insights into the intrinsic properties of lung structure in OI patients and shedding light on the potential underlying mechanisms of respiratory complications.

In conclusion, it appeared possible to show airway obstruction using FOT in patients with OI, from unobtrusive measurements during quiet breathing. The decrease in resistance at 8 Hz following salbutamol administration indicates the sensitivity of FOT to measure bronchial obstruction and its response to therapy. The technique may also provide information on restrictive lung function, as it provides separate estimates of impedance during both inspiration and expiration. A high inspiratory impedance is a possible sign of restrictive lung disease, whereas a high expiratory impedance has been associated with obstruction of intrathoracic airways. The repeatability of the main FOT parameters was relatively high. Continued exploration of the role of the FOT in monitoring treatment response and disease progression can enhance respiratory health assessment and personalized care for individuals with OI.

## Data availability statement

The original contributions presented in the study are included in the article, further inquiries can be directed to the corresponding author.

## Ethics statement

The studies involving humans were approved by Medical Ethics Review Committee (MERC) of the Amsterdam UMC (Amsterdam, The Netherlands). The studies were conducted in accordance with the local legislation and institutional requirements. The participants provided their written informed consent to participate in this study. Written informed consent was obtained from the individual(s) for the publication of any potentially identifiable images or data included in this article.

## Author contributions

SS: Conceptualization, Methodology, Writing – original draft, Writing – review & editing. SV: Writing – review & editing. DM: Writing – review & editing. PJ: Writing – review & editing. MB: Writing – review & editing. EE: Conceptualization, Supervision, Writing – review & editing. JA: Conceptualization, Data curation, Supervision, Writing – review & editing.
